# 2,3-Dichloro­benzene-1,4-diol

**DOI:** 10.1107/S1600536809014408

**Published:** 2009-04-25

**Authors:** Paul D. Ahn, Roger Bishop, Donald C. Craig, Marcia L. Scudder

**Affiliations:** a School of Chemistry, University of New South Wales, Sydney 2052, Australia

## Abstract

The achiral title compound, C_6_H_4_Cl_2_O_2_, crystallizes with O—H⋯O hydrogen bonding linking mol­ecules into layers. Between layers there are chains of Cl⋯Cl⋯Cl inter­actions with alternating distances of 3.274 (2) and 3.742 (2) Å. Augmenting this arrangement there are also C—H⋯Cl (2.97 and 3.17 Å) and Cl⋯π (shortest distances 3.40 and 3.54 Å) inter­actions.

## Related literature

For the structures of related dichloronaphthalenediols, see: Ahn *et al.* (1995 ▶, 1996 ▶). For the preparation of the title compound, see: Beddoes *et al.* (1981 ▶).
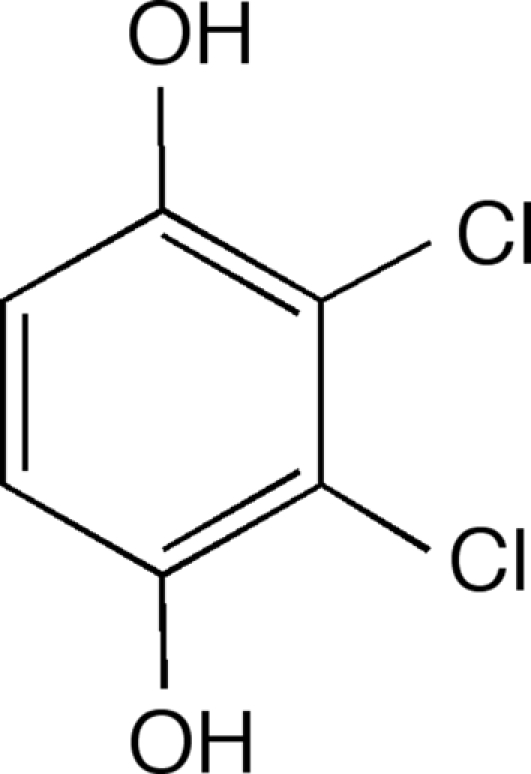


## Experimental

### 

#### Crystal data

C_6_H_4_Cl_2_O_2_
*M* *_r_* = 179.0
Monoclinic, 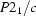

*a* = 4.831 (1) Å
*b* = 11.347 (2) Å
*c* = 12.962 (3) Å
β = 105.94 (1)°
*V* = 683.2 (2) Å^3^
*Z* = 4
Cu *K*α radiation
μ = 8.02 mm^−1^
*T* = 294 K
0.15 × 0.15 × 0.06 mm

#### Data collection

Enraf–Nonius CAD-4 diffractometer
Absorption correction: analytical (de Meulenaer & Tompa, 1965 ▶) *T*_min_ = 0.33, *T*_max_ = 0.63
1449 measured reflections
1290 independent reflections
1187 reflections with *I* > 2σ(*I*)
*R*_int_ = 0.041
1 standard reflections frequency: 30 min intensity decay: 21%

#### Refinement

*R*[*F* ^2^ > 2σ(*F* ^2^)] = 0.034
*wR*(*F* ^2^) = 0.050
*S* = 1.87
1290 reflections
92 parameters
H-atom parameters not refined
Δρ_max_ = 0.42 e Å^−3^
Δρ_min_ = −0.31 e Å^−3^

### 

Data collection: *CAD-4 Manual* (Schagen *et al.*, 1989 ▶); cell refinement: *CAD-4 Manual*; data reduction: local program; program(s) used to solve structure: *SIR92* (Altomare *et al.*, 1994 ▶); program(s) used to refine structure: *RAELS* (Rae, 2000 ▶); molecular graphics: *ORTEP-3* (Farrugia, 1997 ▶) and *CrystalMaker* (CrystalMaker Software, 2005 ▶); software used to prepare material for publication: local programs.

## Supplementary Material

Crystal structure: contains datablocks global, I. DOI: 10.1107/S1600536809014408/bv2117sup1.cif

Structure factors: contains datablocks I. DOI: 10.1107/S1600536809014408/bv2117Isup2.hkl

Additional supplementary materials:  crystallographic information; 3D view; checkCIF report

## Figures and Tables

**Table 1:** Hydrogen-bond geometry (Å, °)

*D*—H⋯*A*	*D*—H	H⋯*A*	*D*⋯*A*	*D*—H⋯*A*
O1—H1*O*1⋯O2^i^	1.00	1.84	2.794 (2)	158
O2—H1*O*2⋯O1^ii^	1.00	1.78	2.778 (2)	172

Symmetry codes: (i) 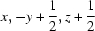; (ii) 
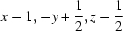
.

**Fig. 1.**
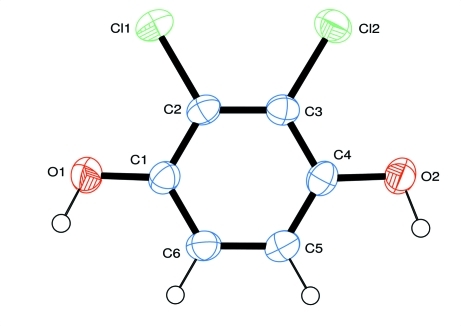
A molecule of the title compound, showing atom labelling. Thermal ellipsoids are drawn at the 50% level.

**Fig. 2.**
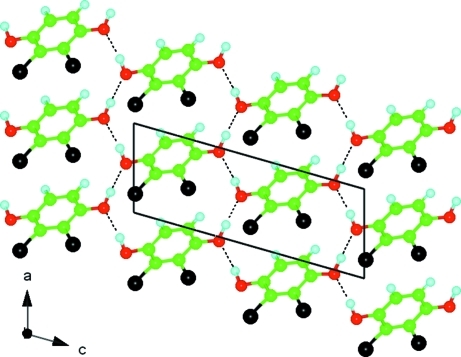
A hydrogen bonded layer. Each molecule participates in two donor and two acceptor hydrogen bonds which are represented as dashed lines.

## supplementary crystallographic information

### Comment

Crystal structures of related dichloronaphthalenediols have been previously reported by us (Ahn *et al.*, 1995, 1996). In the title compound (Fig 1), each molecule participates in four hydrogen bonds, two as donor and two as acceptor, creating a layer structure in the *ac* plane with O1-H1O1···O2-H1O2···O1-H1O1··· chains parallel to *a* (Fig 2, Table 1). Aromatic offset face-face stacking at a distance of 3.5 Å takes place within the layer. Chains of Cl1···Cl1···Cl1 interactions (alternating distances 3.274 (2) and 3.742 (2) Å) which also run parallel to *a* link the layers into a three-dimensional array. In addition there are C5H···Cl2 and C6H···Cl2 (2.97 and 3.17 Å) and Cl2···π interactions (shortest distances 3.40 and 3.54 Å).

### Experimental

2,3-Dichlorobenzene-1,4-diol was prepared as described (Beddoes *et al.*, 1981). ^1^H NMR (300 MHz, d_6_-DMSO) δ 6.79 (s, 2H), 9.71 (s, 2H, –OH); ^13^C NMR (75 MHz, d_6_-DMSO) δ 115.1 (CH), 119.2 (C), 147.1 (C). X-ray quality solvent-free crystals were obtained from benzene solution.

### Refinement

H atoms attached to C were included at calculated positions (C—H = 1.0 Å). The hydroxy hydrogen atoms were located on a difference map, and were then fixed at a position along the OH vector with O—H = 1.0 Å. All hydrogen atoms were refined with isotropic thermal parameters equivalent to those of the atom to which they were bonded.

### Crystal data

**Table:**

C_6_H_4_Cl_2_O_2_	*F*(000) = 360.0
*M**_r_* = 179.0	*D*_x_ = 1.74 Mg m^−^^3^
Monoclinic, *P*2_1_/*c*	Cu *K*α radiation, λ = 1.54184 Å
*a* = 4.831 (1) Å	Cell parameters from 10 reflections
*b* = 11.347 (2) Å	θ = 25–30°
*c* = 12.962 (3) Å	µ = 8.02 mm^−^^1^
β = 105.94 (1)°	*T* = 294 K
*V* = 683.2 (2) Å^3^	Tabular, colourless
*Z* = 4	0.15 × 0.15 × 0.06 mm

### Data collection

**Table:**

Enraf–Nonius CAD-4 diffractometer	*R*_int_ = 0.041
ω–2θ scans	θ_max_ = 70°
Absorption correction: analytical (de Meulenaer & Tompa, 1965)	*h* = −5→0
*T*_min_ = 0.33, *T*_max_ = 0.63	*k* = −13→0
1449 measured reflections	*l* = −15→15
1290 independent reflections	1 standard reflections every 30 min
1187 reflections with *I* > 2σ(*I*)	intensity decay: 21%

### Refinement

**Table:**

Refinement on *F*	0 restraints
*R*[*F*^2^ > 2σ(*F*^2^)] = 0.034	H-atom parameters not refined
*wR*(*F*^2^) = 0.050	*w* = 1/[σ^2^(*F*) + 0.0004*F*^2^]
*S* = 1.87	(Δ/σ)_max_ = 0.008
1290 reflections	Δρ_max_ = 0.42 e Å^−^^3^
92 parameters	Δρ_min_ = −0.31 e Å^−^^3^

### Fractional atomic coordinates and isotropic or equivalent isotropic displacement parameters (Å^2^)

**Table:**

	*x*	*y*	*z*	*U*_iso_*/*U*_eq_	
Cl1	0.78228 (10)	0.11149 (4)	0.49607 (4)	0.0444 (2)	
Cl2	0.54566 (11)	0.00422 (4)	0.26478 (4)	0.0439 (2)	
O1	0.4715 (3)	0.3170 (1)	0.5364 (1)	0.0463 (4)	
O2	0.0408 (3)	0.1224 (1)	0.1331 (1)	0.0404 (4)	
C1	0.3548 (4)	0.2698 (2)	0.4364 (2)	0.0340 (4)	
C2	0.4868 (4)	0.1726 (2)	0.4063 (1)	0.0328 (4)	
C3	0.3797 (4)	0.1238 (2)	0.3049 (2)	0.0319 (4)	
C4	0.1366 (4)	0.1719 (2)	0.2334 (1)	0.0334 (4)	
C5	0.0028 (4)	0.2679 (2)	0.2647 (2)	0.0363 (4)	
C6	0.1126 (4)	0.3174 (2)	0.3651 (2)	0.0363 (4)	
H1O1	0.3196	0.3578	0.5622	0.046	
H1O2	−0.1619	0.1499	0.1026	0.040	
HC5	−0.1745	0.3018	0.2143	0.036	
HC6	0.0168	0.3879	0.3864	0.036	

### Atomic displacement parameters (Å^2^)

**Table:**

	*U*^11^	*U*^22^	*U*^33^	*U*^12^	*U*^13^	*U*^23^
Cl1	0.0340 (3)	0.0503 (3)	0.0427 (3)	0.0103 (2)	−0.0001 (2)	0.0039 (2)
Cl2	0.0407 (3)	0.0389 (3)	0.0521 (4)	0.0050 (2)	0.0128 (2)	−0.0066 (2)
O1	0.0360 (8)	0.0620 (9)	0.0367 (7)	0.0060 (6)	0.0030 (6)	−0.0131 (7)
O2	0.0365 (7)	0.0492 (8)	0.0324 (7)	−0.0001 (6)	0.0044 (6)	−0.0040 (6)
C1	0.0291 (9)	0.0403 (9)	0.0316 (9)	−0.0004 (7)	0.0068 (7)	−0.0004 (7)
C2	0.0251 (8)	0.0374 (9)	0.0352 (9)	0.0018 (7)	0.0072 (7)	0.0040 (7)
C3	0.0281 (9)	0.0318 (8)	0.037 (1)	0.0001 (6)	0.0101 (7)	0.0009 (7)
C4	0.0302 (9)	0.0386 (9)	0.0306 (9)	−0.0049 (7)	0.0070 (7)	0.0018 (7)
C5	0.0314 (9)	0.040 (1)	0.036 (1)	0.0038 (7)	0.0061 (7)	0.0051 (8)
C6	0.031 (1)	0.039 (1)	0.039 (1)	0.0056 (7)	0.0097 (8)	0.0020 (8)

### Geometric parameters (Å, °)

**Table:**

Cl1—C2	1.721 (2)	C1—C6	1.386 (2)
Cl2—C3	1.727 (2)	C2—C3	1.389 (3)
O1—C1	1.372 (2)	C3—C4	1.392 (3)
O1—H1O1	1.000	C4—C5	1.383 (3)
O2—C4	1.374 (2)	C5—C6	1.383 (3)
O2—H1O2	1.000	C5—HC5	1.000
C1—C2	1.383 (3)	C6—HC6	1.000
			
C1—O1—H1O1	110.4	C2—C3—C4	120.1 (2)
C4—O2—H1O2	106.6	O2—C4—C3	118.2 (2)
O1—C1—C2	118.4 (2)	O2—C4—C5	122.4 (2)
O1—C1—C6	122.1 (2)	C3—C4—C5	119.4 (2)
C2—C1—C6	119.5 (2)	C4—C5—C6	120.4 (2)
Cl1—C2—C1	119.5 (1)	C4—C5—HC5	119.8
Cl1—C2—C3	120.3 (1)	C6—C5—HC5	119.8
C1—C2—C3	120.3 (2)	C1—C6—C5	120.3 (2)
Cl2—C3—C2	121.0 (1)	C1—C6—HC6	119.8
Cl2—C3—C4	118.9 (1)	C5—C6—HC6	119.8

### Hydrogen-bond geometry (Å, °)

**Table:**

*D*—H···*A*	*D*—H	H···*A*	*D*···*A*	*D*—H···*A*
O1—H1O1···O2^i^	1.00	1.84	2.794 (2)	158
O2—H1O2···O1^ii^	1.00	1.78	2.778 (2)	172

Symmetry codes: (i) *x*, −*y*+1/2, *z*+1/2; (ii) *x*−1, −*y*+1/2, *z*−1/2.
